# Similar Spatial Expression of Immune‐Related Proteins in SARS‐CoV‐2 Placentitis and Chronic Histiocytic Intervillositis

**DOI:** 10.1002/eji.202451386

**Published:** 2025-01-16

**Authors:** Michelle Broekhuizen, Marie‐Louise van der Hoorn, Disha Vadgama, Michael Eikmans, Bojou J. Neecke, Johannes J. Duvekot, Pieter Fraaij, Irwin K. M. Reiss, Dana A. M. Mustafa, Lotte E. van der Meeren, Sam Schoenmakers

**Affiliations:** ^1^ Division of Neonatology Department of Neonatal and Pediatric Intensive Care Erasmus MC Rotterdam The Netherlands; ^2^ Division of Pharmacology and Vascular Medicine Department of Internal Medicine Erasmus MC Rotterdam The Netherlands; ^3^ Department of Obstetrics and Gynecology Leiden University Medical Center Leiden The Netherlands; ^4^ Department of Pathology Erasmus MC Rotterdam The Netherlands; ^5^ Department of Immunology Leiden University Medical Center Leiden Netherlands; ^6^ Department of Pathology Pathan Rotterdam The Netherlands; ^7^ Department of Obstetrics and Gynecology Erasmus MC Rotterdam The Netherlands; ^8^ Department of Viroscience Erasmus MC Rotterdam The Netherlands; ^9^ Division Infectious Diseases and Immunology Department of Pediatrics Erasmus MC Rotterdam The Netherlands; ^10^ Department of Pathology Leiden University Medical Center Leiden The Netherlands

**Keywords:** apoptosis, chronic histiocytic intervillositis, COVID‐19, myeloid cells, placenta, SARS‐CoV‐2, T‐lymphocytes

## Abstract

Severe acute respiratory syndrome coronavirus 2 (SARS‐CoV‐2) infection in the placenta can lead to fetal distress and demise, characterized by severe trophoblast necrosis, chronic histiocytic intervillositis (CHI), and massive perivillous fibrin deposition. We aimed to uncover spatial immune‐related protein changes in SARS‐CoV‐2 placentitis compared with CHI placentas and uncomplicated pregnancies to gain insight into the underlying pathophysiological mechanisms. Placentas were retrospectively collected from cases with SARS‐CoV‐2 placentitis resulting in fetal distress/demise (*n* = 9), CHI (*n* = 9), and uncomplicated term controls (*n* = 9). The expression of 53 immune‐related proteins was quantified using GeoMx Digital Spatial Profiler in three separate compartments: villi (fetal compartment), intervillous space, and decidua (both maternal compartments). Compared with controls, SARS‐CoV‐2 placentitis and CHI both displayed differentially expressed proteins in the intervillous space only, including upregulation of myeloid markers (e.g., CD40, CD11c, CD68, CD163). Specifically, SARS‐CoV‐2 placentitis was associated with reduced expression of multiple apoptotic proteins (e.g., BAD, BIM, BLXL, BCL6). In conclusion, SARS‐CoV‐2 placentitis and CHI are associated with enhanced myeloid cell infiltration into the intervillous space, but not in the decidua and villi. The more prominently reduced apoptosis‐related protein expression in SARS‐CoV‐2 placentitis may lead to an exaggerated immune response, causing acute placental dysfunction and fetal demise.

## Graphical abstract

SARS‐CoV‐2 placentitis and chronic histiocytic intervillositis are associated with massive infiltration of myeloid cells and T cell activation limited to the placental intervillous space, and not in villi or decidual areas. The prominently reduced expression of apoptosis‐related proteins in SARS‐CoV‐2 placentitis could be related to more acute and severe outcomes.

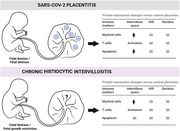

## 1 Introduction

Since the start of the COVID‐19 pandemic, women infected with severe acute respiratory syndrome coronavirus 2 (SARS‐CoV‐2) during pregnancy were reported to have an increased risk for maternal morbidity, mortality and adverse neonatal outcomes [1]. Throughout pregnancy, the placenta acts as a physical and immunological barrier preventing the vertical transmission of viruses to the fetus. Despite rare vertical transmission of SARS‐CoV‐2 during the COVID‐19 pandemic [2, 3], SARS‐CoV‐2 infection of the placenta does occasionally occur and has been associated with fetal distress and demise, even without clinically severe COVID‐19 symptoms in the pregnant woman [3, 4, 5, 6].

**FIGURE 1:**
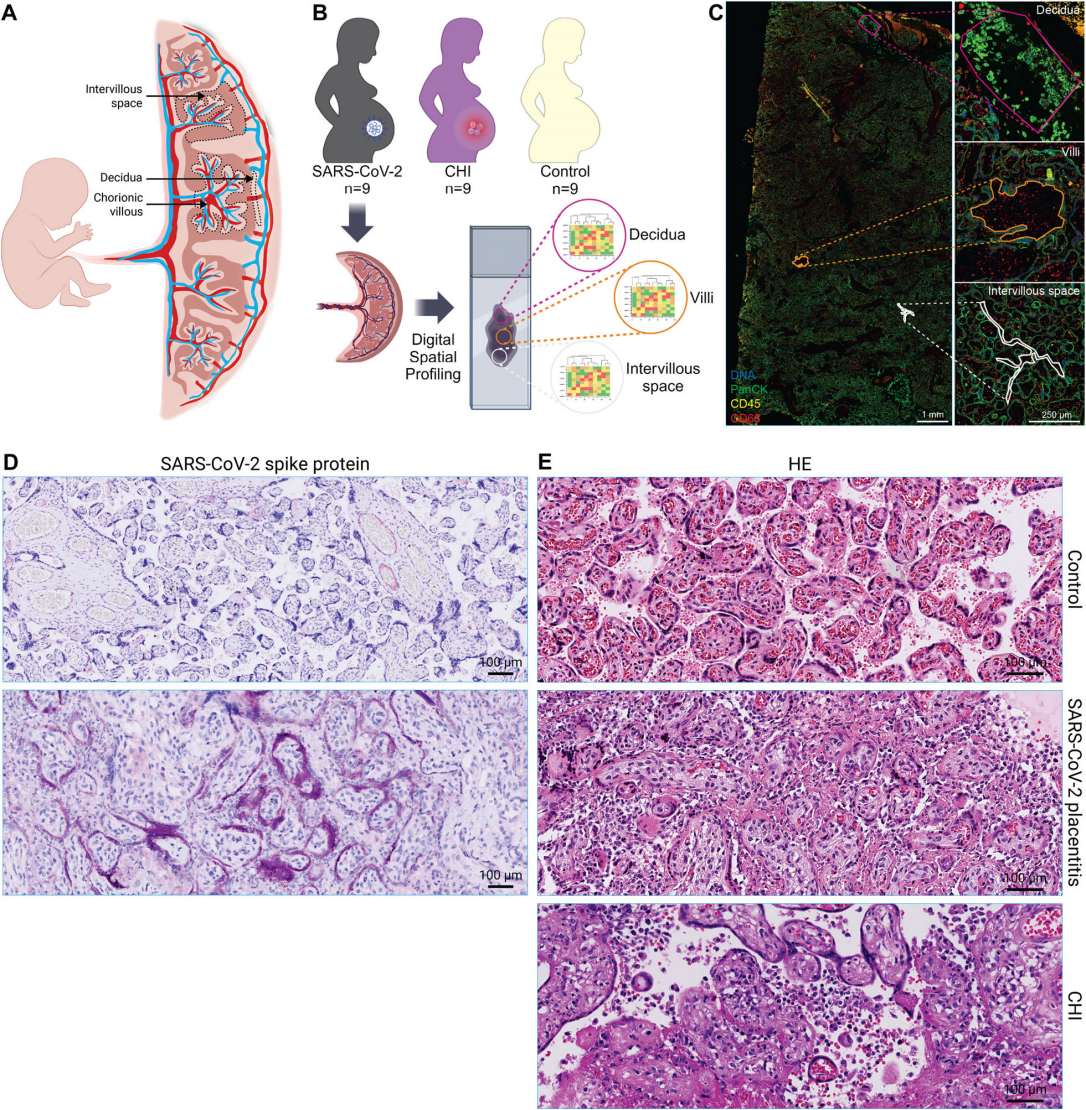
Study overview. (A) Illustration of the placenta displaying the chorionic villi (fetal tissue), intervillous space (maternal blood), and decidua (maternal tissue). (B) Schematic overview of the study setup for Digital Spatial Profiling, created with BioRender. (C) Example of the selection of placental compartments for Digital Spatial Profiling using the expression of immunofluorescent morphological markers PanCK to stain the syncytiotrophoblast (green), CD45 to stain immune cells (yellow), and CD68 to stain macrophages (red). (D) Representative immunohistochemistry images (20×) of antibody staining against SARS‐CoV‐2 spike protein (in purple) of a negative control placenta (top), and a confirmed SARS‐CoV‐2 infection in SARS‐CoV‐2 placentitis (bottom). (E) Representative hematoxylin and eosin (HE) immunohistochemistry images (40×) display healthy term control placentas (top), diffuse chronic intervillositis, perivillous fibrin depositions, and trophoblast necrosis in SARS‐CoV‐2 placentitis (middle), and chronic intervillositis and perivillous fibrin deposition in chronic hystiocytic intervillositis (CHI, bottom).

In case of placental SARS‐CoV‐2 infection, the virus infects the syncytiotrophoblast, the lining of the fetal chorionic villi that borders the maternal blood in the intervillous space (Figure 1A) [2, 4, 6, 7]. The placental villi express angiotensin‐converting enzyme 2 (ACE2), an identified docking site for entry of the SARS‐CoV‐2 virus [8]. Local placental SARS‐CoV‐2 infection induces severe histopathological alterations, including chronic intervillositis, perivillous fibrin deposition, and trophoblast necrosis, and the combination of these findings has been defined as SARS‐CoV‐2 placentitis [9]. As these histopathological changes can cover more than 50% of the placental tissue [10], SARS‐CoV‐2 placentitis almost inevitably leads to placental insufficiency, hampering maternal–fetal exchange, leading to fetal distress, and eventually fetal demise. Although recently SARS‐CoV‐2 infection during pregnancy was associated with transcriptomic changes in the placental villi [11] and the SARS‐CoV‐2 spike protein was shown to induce the release of pro‐inflammatory cytokines and chemokines by trophoblasts in culture [12], the exact cascade of events leading to SARS‐CoV‐2 placentitis remains unclear.

Part of the histopathological findings of SARS‐CoV‐2 placentitis, including chronic intervillositis and perivillous fibrin deposition, are also found in classical/idiopathic chronic histiocytic intervillositis of unknown etiology (CHI). CHI is a placental disease associated with recurrent pregnancy loss, severe fetal growth restriction and fetal demise. CHI has been observed in concurrence with viral and bacterial placental infections, but also in the absence of pathogens when it is denoted as noninfectious CHI of unknown etiology. Despite its unknown etiology, histopathological similarities between CHI and rejected transplanted donor organs suggest an immune‐related condition (or immune‐CHI) [13], Although women who experienced SARS‐CoV‐2 placentitis seem not at increased risk of recurrence [14], immune‐CHI has a recurrence risk ranging between 30% and 100% [15, 16].

Here, we aimed to study which immune alterations are associated with SARS‐CoV‐2 placentitis compared with placentas with CHI and normal term placentas in three different but equally important placental compartments: chorionic villi (fetal tissue), intervillous space (maternal blood), and decidua (maternal tissue, Figure 1B). We applied spatial immune profiling to discriminate between these compartments and investigate distinct expression patterns of proteins related to immune cell repertoire, immune activation status, and cell death, thereby covering the immune‐related hallmarks of SARS‐CoV‐2 placentitis and CHI (Figure 1C). Gaining insight into the immune changes in SARS‐CoV‐2 placentitis and comparing them to CHI of unknown etiology could aid in understanding the pathophysiological pathways that result in impaired placental function.

## 2 Results

### 2.1 Patient Characteristics

The cohort consisted of 27 placentas divided into three groups: nine with confirmed SARS‐CoV‐2 placentitis, nine with CHI of unknown etiology, and nine controls from uncomplicated term pregnancies (Figure 1B). The clinical outcomes are described in Supporting Information Table S1. The SARS‐CoV‐2 placentitis group included two cases of fetal demise, and seven emergency caesarean sections due to fetal distress (median gestational age (GA): 29 weeks + 1 day). In the CHI group, four fetuses suffered from fetal distress and five fetuses were growth‐restricted (median GA 33 weeks + 3 days). The control group consisted of nine women with uncomplicated pregnancies who delivered at term (median GA: 37 weeks + 5 days).

Histopathological analysis confirmed that all placentas with SARS‐CoV‐2 placentitis had a confirmed SARS‐CoV‐2 infection (Figure 1D), and presented diffuse chronic intervillositis, perivillous fibrin depositions, and trophoblast necrosis, while the control group did not harbor any significant histopathological alterations (Figure 1E). Placentas with CHI displayed an immunological response with infiltration of maternal macrophages, chronic intervillositis, and perivillous fibrin depositions without trophoblast necrosis (Figure 1E).

To study differences in immune‐related proteins, all placental slides were imaged for spatial immune profiling, which included the selection of specific areas of interest, chorionic villi (fetal tissue), intervillous space (maternal blood), and decidua (maternal tissue) using the expression of the morphological markers pan‐cytokeratin (PanCK) to stain syncytiotrophoblast, CD45 to stain immune cells, and CD68 to stain macrophages (Figure 1C).

### 2.2 SARS‐CoV‐2 Placentitis and Chronic Histiocytic Intervillositis Placentas Display Similar Changes in Expression of Immune‐Related Proteins in the Intervillous Space

#### 2.2.1 SARS‐CoV‐2 versus Controls

**FIGURE 2:**
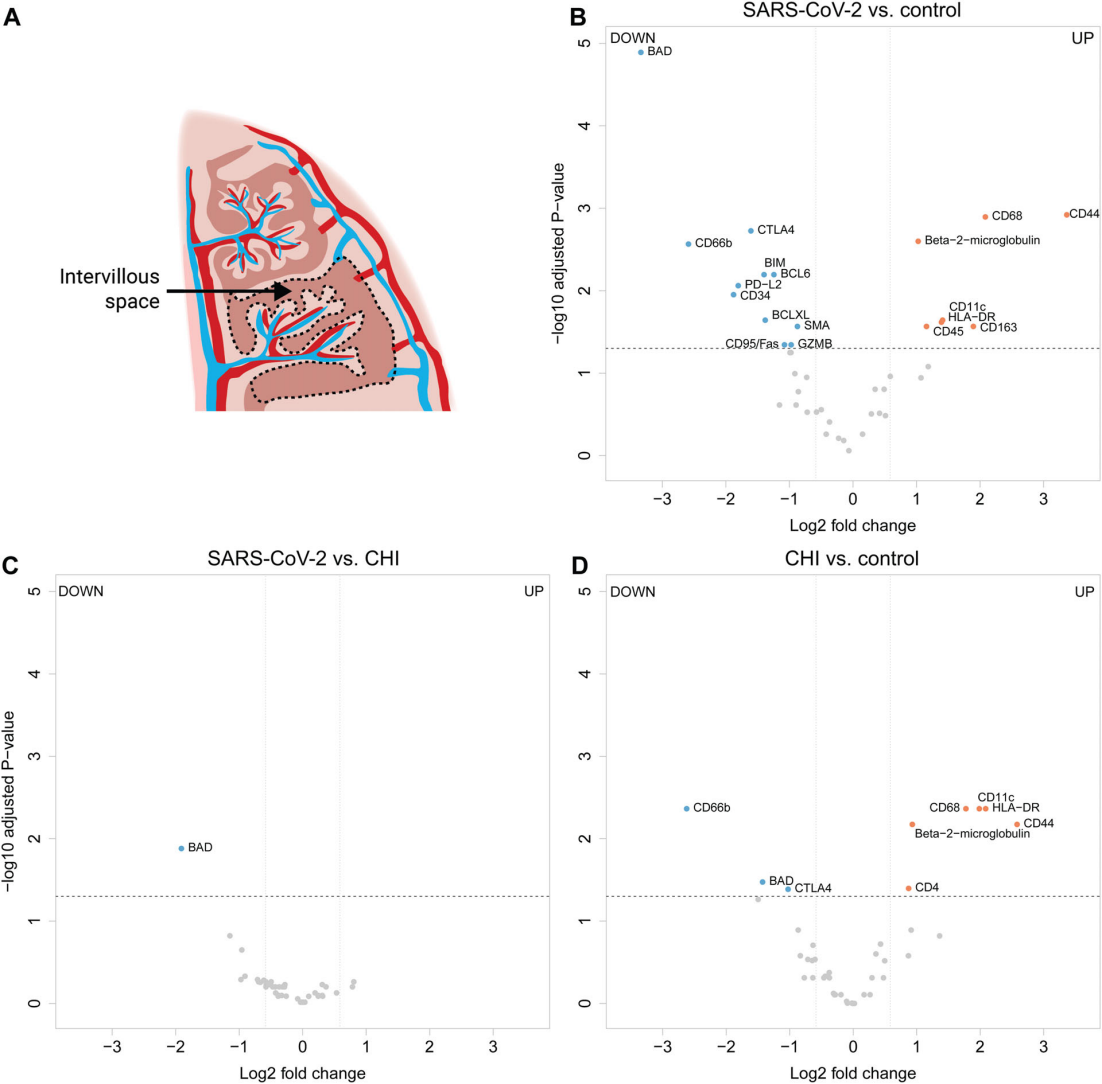
Changes in protein expression in the intervillous space of SARS‐CoV‐2 placentitis, chronic histiocytic intervillositis (CHI), and control placentas. (A) Schematic illustration of the intervillous space. (B) Volcano plot of differentially expressed proteins between SARS‐CoV‐2 placentitis and control placentas. (C) Volcano plot of differentially expressed proteins between chronic histiocytic intervillositis (CHI) and SARS‐CoV‐2 placentitis. (D) Volcano plot of differentially expressed proteins between CHI and control placentas. Proteins were regarded as differentially expressed when adjusted *p*‐value was lower than 0.05 and linear fold change >1.5. Data are representative of three replicate areas in the intervillous space of nine SARS‐CoV‐2 placentitis, nine CHI, and nine control placentas.

Comparing SARS‐CoV‐2 placentitis with controls, we identified statistically significant differences in the expression of 18 proteins in the intervillous space (Figure 2B; Table 1). Of these, 11 proteins were lower expressed in placentas with SARS‐CoV‐2 placentitis compared with controls among which the anti‐apoptotic protein BCL2 associated agonist of cell death (BAD), neutrophil marker CD66b, and hematopoietic marker CD34 displayed the largest fold change.

**TABLE 1:** Changes in protein expression in the intervillous space of placentas with SARS‐CoV‐2 placentitis and chronic histiocytic intervillositis (CHI) compared with each other and with controls.

		SARS‐CoV‐2 vs. control			SARS‐CoV‐2 vs. CHI			CHI vs. control		
Protein	Protein group membership(s)	FC (log2)	*p*‐value	Adj. *p*‐value	FC (log2)	*p*‐value	Adj. *p*‐value	FC (log2)	*p*‐value	Adj. *p*‐value
CD34	Hematopoietic	−**1.88**	**0.003**	**0.011**	−0.38	0.537	0.796	−**1.50**	**0.013**	0.055
CD45	Total immune	**1.16**	**0.010**	**0.027**	0.24	0.594	0.799	**0.91**	**0.034**	0.128
CD68	Myeloid: macrophage	**2.08**	**0.000**	**0.001**	0.31	0.559	0.799	**1.78**	**0.000**	**0.004**
CD163	Myeloid: macrophage, M2 macrophage	**1.89**	**0.009**	**0.027**	0.53	0.482	0.744	**1.36**	**0.046**	0.151
CD11c	Myeloid: dendritic cell	**1.41**	**0.007**	**0.023**	−0.58	0.225	0.590	**1.99**	**0.000**	**0.004**
CD66b	Myeloid: neutrophil	−**2.59**	**0.000**	**0.003**	0.03	0.937	0.964	−**2.62**	**0.000**	**0.004**
CD40	Myeloid: activation	**1.18**	**0.043**	0.083	0.31	0.654	0.815	0.87	0.105	0.264
PD‐L1	Myeloid: activation, immune checkpoint	−0.50	0.206	0.277	0.37	0.365	0.628	−**0.87**	**0.036**	0.128
PD‐L2	Antigen presentation: immune checkpoint, T cells	−**1.80**	**0.002**	**0.009**	−0.97	0.083	0.513	−0.83	0.110	0.264
HLA‐DR	Antigen presentation: MHC class II, activation	**1.39**	**0.008**	**0.024**	−0.69	0.146	0.546	**2.09**	**0.000**	**0.004**
Beta‐2‐microglobulin	Antigen presentation: nucleated cells	**1.03**	**0.000**	**0.003**	0.10	0.705	0.819	**0.93**	**0.001**	**0.007**
CD4	T cells: Th cells, myeloid	0.59	0.061	0.109	−0.29	0.350	0.628	**0.87**	**0.007**	**0.040**
CD44	T cells: cell adhesion	**3.36**	**0.000**	**0.001**	0.78	0.297	0.628	**2.58**	**0.001**	**0.007**
CTLA‐4	T cells: activation, regulation, immune checkpoint	−**1.61**	**0.000**	**0.002**	−0.58	0.125	0.546	−**1.03**	**0.009**	**0.041**
GZMB	Cytotoxicity	−**0.97**	**0.020**	**0.045**	−0.37	0.354	0.628	−0.60	0.136	0.292
GZMA	Cytotoxicity	−**0.99**	**0.026**	0.056	−0.53	0.215	0.590	−0.46	0.285	0.488
BAD	Apoptosis: pro‐apoptosis	−**3.34**	**0.000**	**0.000**	−**1.91**	**0.001**	**0.013**	−**1.43**	**0.005**	**0.034**
BIM	Apoptosis: pro‐apoptosis	−**1.40**	**0.001**	**0.006**	−**0.96**	**0.021**	0.224	−0.44	0.261	0.467
CD95/Fas	Apoptosis: pro‐apoptosis	−**1.08**	**0.020**	**0.045**	−0.43	0.327	0.628	−0.65	0.147	0.301
BCLXL	Apoptosis: anti‐apoptosis	−**1.38**	**0.007**	**0.023**	−0.67	0.167	0.554	−0.71	0.135	0.292
BCL6	Apoptosis: anti‐apoptosis	−**1.24**	**0.001**	**0.006**	−0.61	0.097	0.522	−0.63	0.069	0.196
SMA	Smooth muscle cells	−**0.87**	**0.011**	**0.027**	−0.50	0.139	0.546	−0.38	0.226	0.422
Fibronectin	Fibroblasts	−**0.97**	**0.027**	0.056	−**1.15**	**0.011**	0.151	0.17	0.674	0.783

*Note*: FC indicates fold change and is depicted as median value; statistically significant results are depicted in bold and obtained through linear mixed models with Benjamin–Hochberg multiple testing adjustment (Adj. *p*‐value).

#### 2.2.2 SARS‐CoV‐2 versus CHI

SARS‐CoV‐2 placentitis and CHI placentas show overall resemblances in expression patterns of immune‐related proteins. Comparing both types of placentas, SARS‐CoV‐2 placentitis presented only one differentially expressed protein in the intervillous space: a decreased expression of the pro‐apoptotic marker BAD (log2 fold changes [FC]: −1.9, *p* = 0.013; Figure 2C; Table 1).

#### 2.2.3 CHI versus Control

Comparing CHI with control placentas, nine differentially expressed proteins were identified within the intervillous space only. Protein expression alterations overlapped with changes observed between SARS‐CoV‐2 placentas and control placentas (Figure 2D; Table 1).

There were no differences in expression of immune‐related proteins in either the villi or decidua in between any of the groups.

### 2.3 Similar Enhanced Expression of Myeloid Immune Cell Markers in the Intervillous Space of SARS‐CoV‐2 Placentitis and Chronic Histiocytic Intervillositis Placentas

#### 2.3.1 SARS‐CoV‐2 versus Controls

**FIGURE 3:**
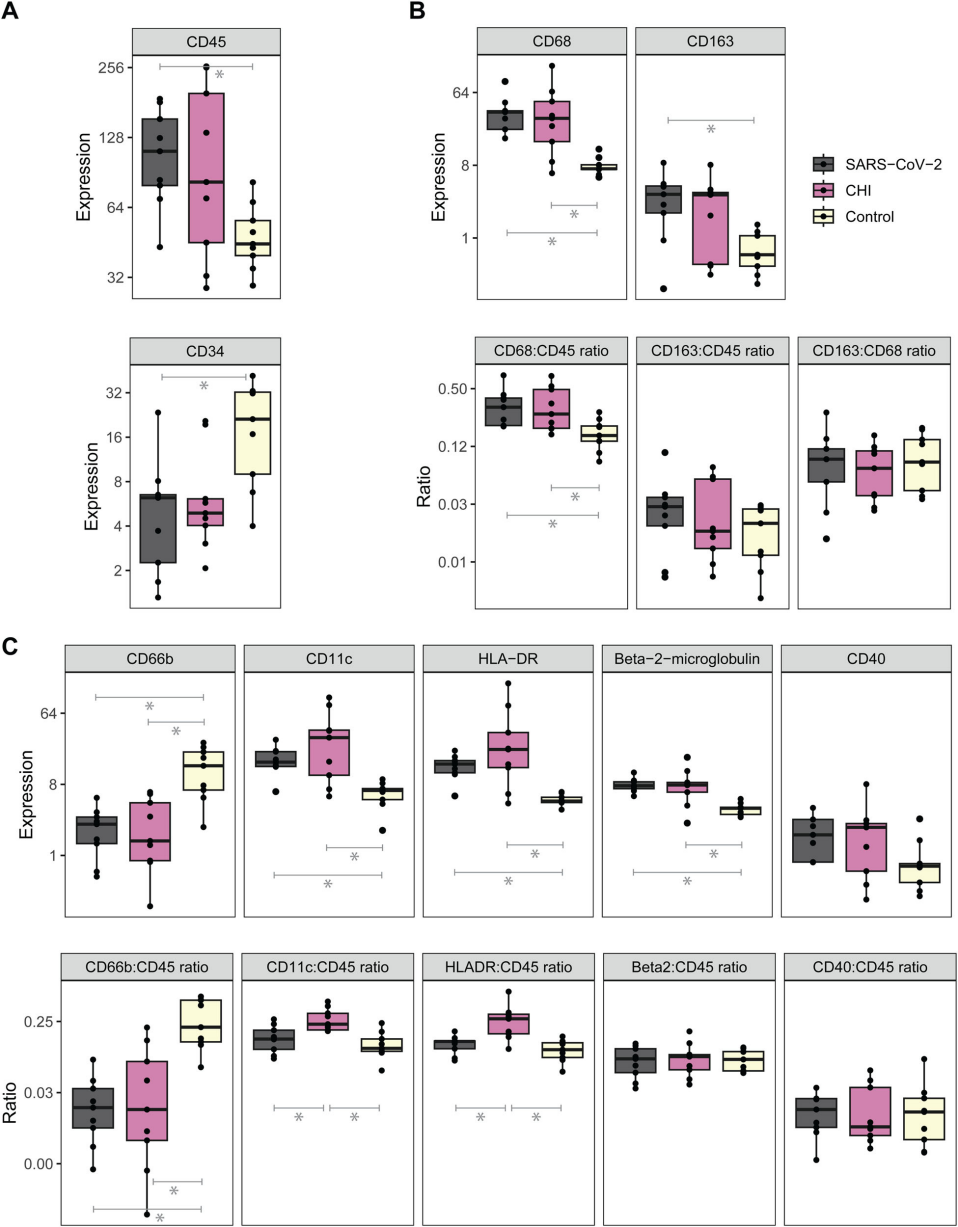
The expression of myeloid‐associated proteins intervillous space of SARS‐CoV‐2 placentitis, chronic histiocytic intervillositis (CHI), and control placentas. (A) Expression of immune cell (CD45) and hematopoietic (CD34) marker. (B) Expression of general macrophage (CD68) and M2‐like macrophage (CD163) marker and the ratio between these two markers. (C) Expression of neutrophil (CD66b), dendritic cell (CD11c), antigen presentation (HLA‐DR, Beta‐2‐microglobulin), and myeloid activation (CD40) markers and their ratios. The points depict the geometric mean expression of three replicate areas in the intervillous space of nine SARS‐CoV‐2 placentitis, nine CHI, and nine control placentas. The expressions and ratios were statistically compared between the groups using linear mixed models with Benjamin–Hochberg correction on the original data.

The analysis of immune cell type markers in the intervillous space revealed elevated expression of general immune cell marker CD45 (log2 FC: 1.2, *p* = 0.027) in SARS‐CoV‐2 placentitis compared with controls, suggesting enhanced immune cell infiltration, while the hematopoietic marker CD34 was decreased (log2 FC: −1.9, *p* = 0.011; Figure 3A). The resulting immune infiltrate mainly comprised of myeloid cells, specifically macrophages (CD68 & CD163, Figure 3B) and antigen‐presenting cells (CD11c, HLA‐DR, Beta‐2‐microglobulin), while the neutrophil marker CD66b was decreased (log2 FC: −2.6, *p* = 0.003; Figure 3C). To uncover changes in the immune cell balance, the expression of immune cell markers was normalized against CD45. The immune infiltrate in the intervillous space of placentas with SARS‐CoV‐2 placentitis compared with controls consisted of relatively more macrophages (CD68:CD45 ratio log2 FC: 0.9, *p* = 0.020) and fewer neutrophils (CD66b:CD45 ratio log2 FC: −3.7, *p* = 0.002), while the proportion of M2 macrophages to the total macrophage population, the CD163:CD68 ratio, was not different (Figure 3B,C).

#### 2.3.2 SARS‐CoV‐2 versus CHI

Although the absolute expression of myeloid immune cell markers was not different between SARS‐CoV‐2 placentitis and CHI placentas, the CD11c:CD45 and HLADR:CD45 ratios were exclusively elevated in CHI placentas (Figure 3C). This suggests that there is a higher proportion of antigen‐presenting dendritic cells in the intervillous space in cases with CHI.

#### 2.3.3 CHI versus Control

Similar to the findings within SARS‐CoV‐2‐placentitis, CHI placentas displayed trends toward downregulation of the hematopoietic stem cell marker CD34 (log2 FC: −1.5, *p* = 0.055), and upregulation of the total immune cell marker CD45 (log2 FC: 0.9, *p* = 0.128), together with increased expression of myeloid cell markers (Figure 3A–C; Table 1).

### 2.4 More Pronounced Changes in the Expression of Apoptotic Markers in the Intervillous Space with SARS‐CoV‐2 Placentitis

#### 2.4.1 SARS‐CoV‐2 versus Control

**FIGURE 4:**
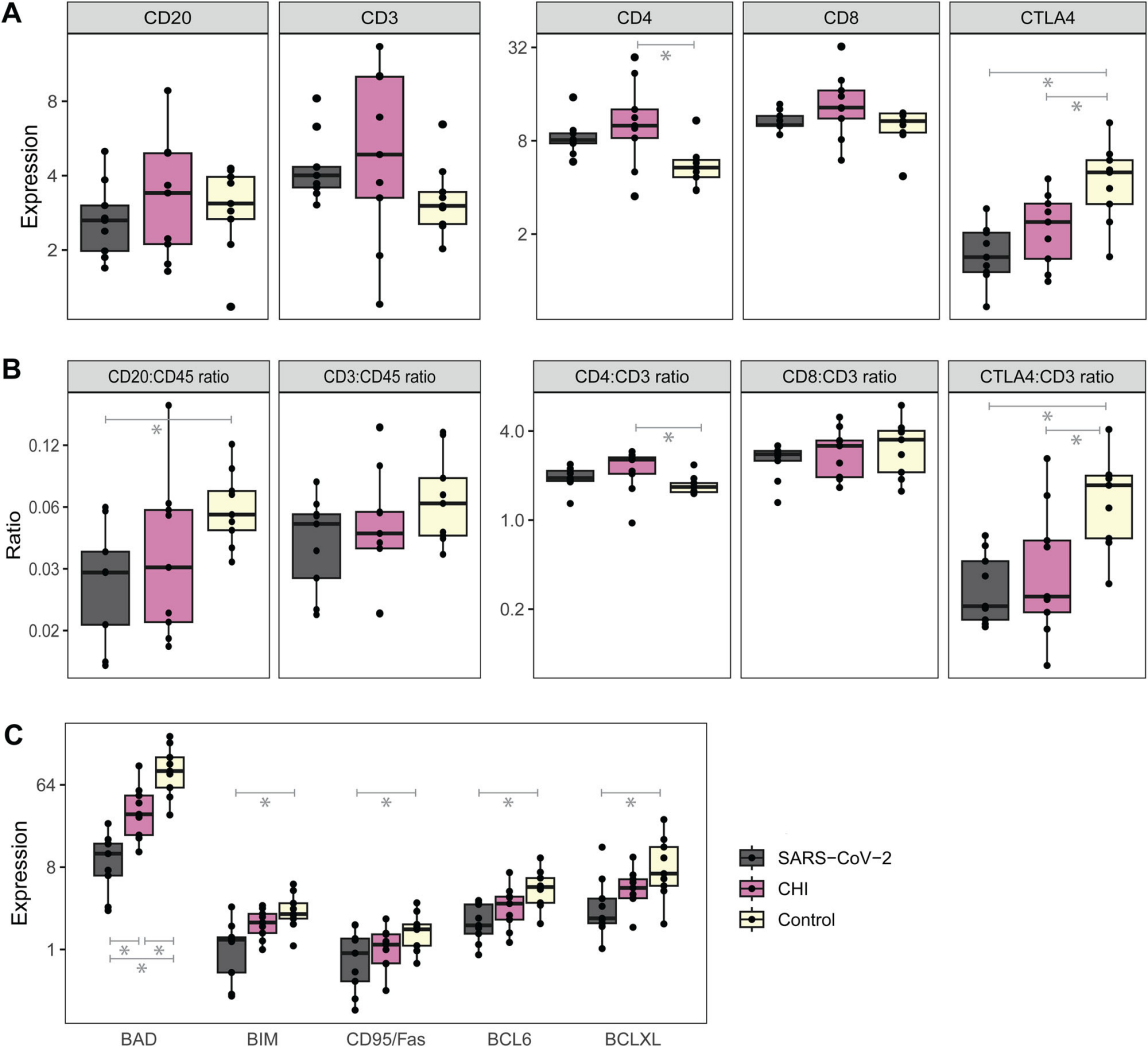
The expression of markers of T cell activation and apoptosis in the intervillous space of SARS‐CoV‐2 placentitis, chronic histiocytic intervillositis (CHI), and control placentas. (A) The expression of the B cell marker CD20, T cell marker CD3, T cell subtypes CD4 and CD8, and CTLA‐4. (B) The ratios of these markers to CD45 and CD3. (C) The expression of pro‐ and anti‐apoptotic proteins. The points depict the geometric mean expression of three replicate areas in the intervillous space of nine SARS‐CoV‐2 placentitis, nine CHI, and nine control placentas. The expressions and ratios were statistically compared using linear mixed models with Benjamin‐Hochberg correction on the original data.

The extent of infiltration of adaptive immune cells (general B and T cell markers) in the intervillous space did not differ between SARS‐CoV‐2 placentitis and control placentas as CD20 and CD3 were similarly expressed. However, reduced expression of T cell activation marker CTLA‐4 (log2 FC: −1.6, *p* = 0.002) suggests a difference in T cell activation (Figure 4A,B).

Pathway analysis showed downregulation of apoptotic proteins in SARS‐CoV‐2 placentitis compared with controls as displayed in Figure 4C. This figure also illustrates a relatively high expression of the pro‐apoptotic marker BAD in comparison with the other apoptotic markers under healthy circumstances, which is most significantly decreased in SARS‐CoV‐2 placentitis (log2 FC: −3.3, *p* < 0.001; Table 1).

#### 2.4.2 SARS‐CoV‐2 versus CHI

There were no differences in the expression of B‐ and T‐cell markers in the intervillous space between SARS‐CoV‐2 placentitis and CHI placentas. However, the expression of BAD was significantly more reduced in SARS‐CoV‐2 placentitis (Figure 4C; Table 1).

#### 2.4.3 CHI versus Control

CHI placentas displayed reduced expression of T cell activation marker CTLA‐4 (log2 FC: −1.0, *p* = 0.023), similar to placentas with SARS‐CoV‐2 placentitis. Although general T cell marker CD4 was only elevated in CHI versus control placentas (log2 FC: 0.9, *p* = 0.040; Figure 4A,B).

Besides the downregulation of BAD (log2 FC: −1.4, *p* = 0.034), CHI placentas did not display any changes in the expression of apoptotic proteins, in contrast with data from SARS‐CoV‐2 placentitis. In addition, BAD was downregulated less significantly than in SARS‐CoV‐2 placentitis versus control placentas (Figure 4C).

## 3 Discussion

SARS‐CoV‐2 placentitis presents with severe histopathological features in the placenta that interfere with placental function and it has been associated with fetal distress and demise. Although SARS‐CoV‐2 placentitis was reported throughout the whole pandemic [3, 4, 17], particularly during dominance of the SARS‐CoV‐2 B1.617.2 (Delta) variant, the risk of stillbirth was significantly increased from 0.63% to 2.70% [18]. The current study uncovered that compared with control placentas, placentas with SARS‐CoV‐2 placentitis display significant and gross alterations in the immune infiltrate only within the intervillous space, likely hampering the essential maternal–fetal exchange of oxygen and nutrients. These alterations were grossly similar to those in the intervillous space of placentas with CHI of unknown etiology and included upregulation of myeloid markers, while SARS‐CoV‐2 placentitis was associated with more prominently reduced expression of apoptosis‐related proteins. Villi and decidual areas of SARS‐CoV‐2 and CHI placentas did not show any differentially expressed immune‐related proteins compared with controls.

The more pronounced changes in expression of apoptosis‐related proteins, the extent of placental lesions in SARS‐CoV‐2 placentitis, and a relatively short duration between the moment of SARS‐CoV‐2 infection and onset of clinical symptoms in the fetus [10], point toward more rapid disease progression compared with CHI. The rapid disease progression and acute onset of extensive placental damage allow no time for fetal or placental adaptation, providing an explanation for the sudden occurrence of fetal distress and demise without prior indication of fetal problems that we and others have observed clinically [10]. In line with this acute onset, the placental weight is within the normal range in SARS‐CoV‐2 placentitis [6]. Slower progression of placental lesions and less involvement of apoptosis in CHI enable the fetus to adapt to a slowly decreasing perfusion and gas–nutrient exchange. These cases typically present with a lower placental weight in combination with clinical features of fetal growth restriction or fetal distress [19]. Previous evidence from CHI suggests that widespread severe intervillositis with massive fibrinoid depositions is associated with a worse perinatal prognosis, in contrast with more focal and less widespread tissue involvement [20]. Placental infection by SARS‐CoV‐2 thus seems to lead to a more rapid and drastic immune response in the intervillous space, with subsequent necrosis and fibrin formation, obstructing maternal blood flow through the intervillous space. We speculate that this ultimately causes an increasingly impaired exchange of oxygen, nutrients, and waste products between the mother and the fetus, although this study provides no direct measures for oxygen or nutrient exchange, and this remains subject for future research

Particularly, placentas with SARS‐CoV‐2 placentitis display extended areas of necrosis and reduced expression of apoptosis‐related proteins which could be attributed to the virus itself. SARS‐CoV‐2 was shown to colocalize with the extended areas of necrosis and regulate apoptosis most notably via the BCL‐2 protein family [21]. Also, the present data displayed reduced expression of two BCL‐2 protein family members, BCLXL and BCL6, exclusively in SARS‐CoV‐2 placentitis. Dysregulation of the apoptotic processes may explain the massive amount of tissue destruction that is present in SARS‐CoV‐2 placentitis.

The expression patterns of immune cell‐related proteins suggest enhanced infiltration of immune cells, mainly of the myeloid subtype, in placentas with SARS‐CoV‐2 placentitis and CHI in the intervillous space only. These differentially expressed immune cell markers largely overlap with currently identified general immune alterations in COVID‐19 disease, including upregulation of HLA‐DR, CD11c, and CD163 on circulating monocytes [22]. Specifically looking at macrophages, our data shows increased expression of both CD68 and CD163 in SARS‐CoV‐2 placentitis. Although our protein panel did not contain any specific pro‐inflammatory M1 macrophage markers, under the assumption that most macrophages are either of the CD163‐expressing M2 subtype or the CD163 negative M1 subtype, the absence of a change in the CD163:CD68 ratio data suggests that both M1 and M2 macrophages infiltrate the intervillous space with SARS‐CoV‐2 placentitis.

Interestingly, CD163‐enriched macrophages were reported to have a pro‐fibrotic phenotype and accumulate in the lungs specifically in areas of fibrotic tissue remodeling in patients with severe COVID‐19‐associated acute respiratory distress syndrome [23]. Infiltration of this profibrotic macrophage subtype into the intervillous space most likely contributes to diffuse fibrinoid deposition in SARS‐CoV‐2 placentitis [4]. Although severe COVID‐19 disease was also associated with the appearance of CD66b^+^ proliferative neutrophils in blood [22], we observed a decreased CD66b expression in the intervillous space with SARS‐CoV‐2 placentitis. As the neutrophil proportion was also highly variable in lung autopsy tissue of severe COVID‐19 patients [23], the number of neutrophils may not necessarily be related to disease progression at the tissue level.

Although changes in the expression of proteins related to adaptive immune cells are minor, we observed an elevated expression of T cell marker CD4 in the intervillous space of CHI placentas and a similar trend in placentas with SARS‐CoV‐2 placentitis. Moreover, both SARS‐CoV‐2 placentitis and CHI placentas displayed reduced expression of the inhibitory T cell receptor CTLA‐4 and concomitantly increased expression of CD44. Although CD44 is not strictly limited to T cells. These data seem distinct from COVID‐19 in general, which has been associated with an enrichment of CD3^+^ T cells in the lungs and an increase in activated and proliferating T helper and CD8^+^ T cells in blood [24]. The altered T cell activation patterns in SARS‐CoV‐2 placentitis and CHI do, however, seem similar to those in graft‐versus‐host disease, which was shown to be potentially triggered by an active viral infection [25]. Under these conditions, the virus may activate allo‐reactive CD4^+^ T cells that can attack nonhematopoietic cells expressing MHC class II molecules as a consequence of inflammatory conditions created by the antiviral immune response [25], potentially inducing a self‐directed destructive process. We believe that this condition may mimic hemophagocytic lymphohistiocytosis at the level of the placenta, with local hyperinflammation, dysregulation of the immune system and a local cytokine storm resulting in necrosis within the placenta. It would be interesting for future studies to determine whether such a local elevation in cytokines levels occurs in SARS‐CoV‐2 placentitis. This may explain how a certain (viral) trigger in the placenta induces a local inflammatory cascade in the intervillous space, leading to infiltration of antigen‐presenting cells, T cell activation, massive perivillous fibrin deposition and a widespread apoptotic cascade.

The overlapping immune alterations between SARS‐CoV‐2 placentitis and CHI suggest that, although having different aetiologies, the resulting immunological mechanisms are similar. For CHI, two potential aetiologies have been proposed: (1) infection by an unrecognized agent, and (2) a graft versus host‐like immune rejection [19, 26, 27, 28, 29]. Data from the current study suggest that both placental SARS‐CoV‐2 infection and CHI involve a certain type of self‐directed tissue destruction.

This study found no immune alterations in the fetal villi and maternal decidua, suggesting spatial segregation of immunological changes between the villi, decidua, and intervillous space. Yet, changes in the maternal intervillous space may still affect the function of fetal villi, as recently maternal SARS‐CoV‐2 infection was associated with transcriptional changes in trophoblasts and the villous core, mainly related to vasodilation, oxidative‐ and placental stress even in absence of placental SARS‐CoV‐2 infection [11]. Moreover, reduced expression of CD34 in the intervillous space of placentas with SARS‐CoV‐2 placentitis and CHI may originate from changes inside the villi. Although CD34 is mainly known as a hematopoietic marker, CD34 in the intervillous space is mainly of fetal origin and expressed by endothelial and non‐hematopoietic lineages [30]. A lower CD34 expression in the intervillous space may thus be due to reduced migration of CD34‐expressing cells from the fetal villi into the intervillous space.

SARS‐CoV‐2 infection and viremia do not seem to affect the placental physical and immunological barrier against hematogenous transmission of viral pathogens. Placental infection by SARS‐CoV‐2 appears mainly limited to the syncytiotrophoblast, which expresses high levels of the SARS‐CoV‐2 receptor ACE2 [10, 31]. Only in a few cases (<12%), SARS‐CoV‐2 was shown to progress further into other fetal placental cell types, including cytotrophoblasts, Hoffbauer cells, and villous endothelial cells [10].

### 3.1 Data Limitations and Perspectives

The application of digital spatial immune profiling in SARS‐CoV‐2 placentitis, CHI and control placentas, allowed us to quantify absolute protein expression in the decidua, intervillous space, and villi separately. Yet, this concerns a general analysis of mRNA expression within each selected area and cannot distinguish changes at single‐cell level, therefore, it remains unclear whether changes in protein expression are due to an increased number of cells, or an increased expression of the marker on the same number of cells. Although we attempted to assemble the protein panels to cover most of the immune processes reported to be involved in SARS‐CoV‐2 infection in previous literature, this study was limited by this predefined panel, and we cannot exclude changes of other proteins and involvement of additional mechanisms such as differences in M1 macrophages and cytokines. Although the average gestational age differed between our groups, it was unfeasible to include controls with uncomplicated pregnancies and a lower gestational age. Yet, the occurrence of protein expression changes only in the intervillous space and their large magnitude makes it unlikely that these are induced (solely) by differences in gestational age. Whether the currently identified changes, such as upregulation of myeloid‐associated proteins, hold promise as biomarkers to help identify patients at risk for SARS‐COV‐2 placentitis or recurrence of CHI depends on their systemic presentation in maternal blood, and remain subject for future research. For those high‐risk patients, pravastatin therapy, acetylsalicylic acid, low molecular weight heparin, or chloroquine may reduce the risk for complications [32, 33].

### 3.2 Conclusion

The current study reveals that severe placental histopathological lesions in SARS‐CoV‐2 placentitis are associated with enhanced infiltration of myeloid cells, T cell activation, and reduced expression of apoptosis‐related proteins specifically in the maternal intervillous space, as the expression of immune‐related proteins in the placental villi and decidua are unchanged. The more prominent alterations in apoptotic processes in SARS‐CoV‐2 placentitis compared with CHI are potentially related to a more rapid disease progression in the placenta, which prevents the fetus from adapting. Although the physical and immunological barriers in the placenta remain intact and prevent the transmission of SARS‐CoV‐2 to the fetus, SARS‐CoV‐2 infection in the syncytiotrophoblast provokes immunological responses in the intervillous space that can be detrimental to placental function and the developing fetus.

## 4 Materials and Methods

### 4.1 Tissue Samples

Samples were collected from the following three groups: placentas from (1) women with a confirmed placental SARS‐CoV‐2 infection who gave birth due to fetal distress/demise, (2) women with histopathologically confirmed CHI of unknown etiology, and (3) women who had an uncomplicated singleton term pregnancy. This study was conducted at the Erasmus MC University Medical Center in Rotterdam, the Netherlands and patients provided written informed consent (MEC‐2020‐0323). SARS‐CoV‐2 placentas and control placentas were collected between March 2020 and December 2021 from the Erasmus MC University Medical Center. The CHI placentas were provided by Leiden University Medical Center, Leiden, the Netherlands, and collected between 2020 and March 2021. To prevent potential unknown or undetected SARS‐CoV‐2 presence in the CHI cases, all CHI placentas were selected from before the COVID‐19 Delta‐variant (before April 2021) which was especially infectious with increased numbers of SARS‐CoV‐2‐placentitis and fetal demise. The pathology study protocol was consistent with international ethical and professional guidelines (the Declaration of Helsinki and the International Conference on Harmonization Guidelines for Good Clinical Practice).

### 4.2 Histopathological Examination

All placentas were macroscopically and microscopically examined according to the Amsterdam criteria by a perinatal pathologist who was blinded for all clinical data and outcome except for gestational age. Gross examination included placental weight (without umbilical cord and membranes) and placental disk dimensions [34]. For microscopic inspection, four main categories of placental pathology included maternal vascular malperfusion, fetal vascular malperfusion, and chronic and acute inflammation.

### 4.3 Immunohistochemistry of SARS‐CoV‐2

The presence of SARS‐CoV‐2 in the placenta was assessed by automated immunohistochemistry using the Discovery ULTRA (Ventana Medical Systems Inc., Oro Valley, AZ, USA). Tissue blocks were cut into 4 µm thick (FFPE) sections. Following deparaffinization and heat‐induced antigen retrieval with CC1 (#950‐500, Ventana) for 32 min, the tissue samples were incubated for 32 min at 37°C with a polyclonal antibody against spike protein (anti‐rabbit SARS‐CoV‐2 Spike, Sabbiotech, College Park, MD, USA). Detection and incubation were done according to the manufacturer's instructions (Ventana). Positive controls were used on every slide.

### 4.4 Digital Spatial Profiling

The spatial expression analysis of immune‐related proteins was performed using the GeoMx Digital Spatial Profiler (DSP) platform from NanoString (Seattle, WA, USA) as described previously [35]. First of all, formalin‐fixed paraffin‐embedded slides of placental tissue were stained, as per manufacturer's instructions, with fluorescently tagged antibody probes to image the morphological markers pan‐cytokeratin (PanCk, NanoString, NBP2‐33200), to stain the syncytiotrophoblast, CD45 (NanoString, NBP2‐34528) to stain immune cells, and CD68 (Abcam, ab224029) to stain macrophages. The slides were also stained with a cocktail of antibodies tagged with unique oligonucleotide barcodes targeting 53 immune‐related proteins, listed in Table S2. These antibodies were included in the predefined nCounter protein panels from NanoString: immune cell profiling core (ICPH10002), human immune activation status (IASH10001), human immune cell typing (ICTH10001), human cell death (CLDH10001), which were carefully chosen to cover most of the immune processes reported to be involved in SARS‐CoV‐2 infection.

The placental slides were imaged on the GeoMx DSP and specific areas of interest were selected by an experienced pathologist using the expression of the morphological markers PanCK, CD45, and CD68. The selected areas were distinguished between the following compartments: chorionic villi (fetal tissue), intervillous space (maternal blood), and decidua (maternal tissue; Figure 1A–C). Per placenta slide, each of the compartments was selected in triplicate to cover heterogeneity within the tissue, resulting in a total of nine selected areas per slide, with average surface areas and nuclei counts of respectively 56,813 µm^2^ and 244 nuclei for the chorionic villi, 33,124 µm^2^ and 117 nuclei for the intervillous space, and 122,849 µm^2^ and 242 nuclei for the decidua. The areas were well‐spaced between replicates to represent the entire surface of the slide. Although the choice of areas was sometimes limited as it was challenging to obtain areas with a sufficient number of cells (e.g., minimal 20 cells in the intervillous space), it was always ensured that the selection covered different zones of the slide. Areas with erythrocyte infiltration (heavy bleeding) were avoided.

Digital spatial profiling quantified the absolute expression of the target proteins in every selected area, by specifically exposing each area to focused UV light using an array of micromirrors, inducing the release of the oligonucleotide barcodes only in the exposed area. These barcodes were collected and used for library preparation and every area was indexed with a different pair of indices so that the resulting library fragments contained a unique molecular identifier and target analyte identifier. These barcodes were counted and traced back to their original areas on the nCounter platform. Quality control analysis and background correction were performed in the GeoMx DSP Analysis Suite (version 2.4.2.2, NanoString) with Rabbit IgG, Mouse IgG2a and Mouse IgG1 as negative controls, and glyceraldehyde 3‐phosphate dehydrogenase (GAPDH) and ribosomal protein S6 as positive controls using the recommended settings; field of view registration >75, binding density 0.1–2.25, positive control normalization 0.3–3, minimum nuclei count >20. The target protein counts were normalized according to the GAPDH and S6 counts in every selected area to correct for differences in tissue area sizes. The expression PanCk was removed from further analyses as it was only used as a morphological marker during the area selection procedure.

Analysis of immune cell subsets was done by dividing the expression of a target protein of interest over the expression of immune marker CD45 (target protein:CD45 ratio), T cell marker CD3 (target protein:CD3 ratio), or macrophage marker CD68 (target protein:CD68 ratio).

### 4.5 Statistical Analysis

Graphs were created in R using the ggplot2 package [36, 37]. Differences in protein expression between the three groups were statistically compared using a Linear Mixed Model with random effects adjustment based on each scan and were corrected for multiple testing through the Benjamin‐Hochberg method in the GeoMx DSP Analysis software (version 2.4.2.2, NanoString). FC are reported as median log2 value with adjusted *p*‐value. Proteins were considered statistically significant and differentially expressed when adjusted *p* was lower than 0.05.

## Author Contributions

Michelle Broekhuizen: Conceptualization, investigation, formal analysis, visualization, and writing–original draft. Marie‐Louise van der Hoorn: Resources and writing–review and editing. Disha Vadgama: Investigation and formal analysis. Michael Eikmans, Bojou J. Neecke, Johannes J. Duvekot, and Pieter Fraaij: Writing–review and editing. Irwin K. M. Reiss: Conceptualization and funding acquisition. Dana A. M. Mustafa: Conceptualization and methodology. Lotte E. van der Meeren: Conceptualization, resources, investigation, and writing–review and editing. Sam Schoenmakers: Conceptualization, resources, writing–review and editing, and funding acquisition. All authors read and approved the final manuscript.

## Conflicts of Interest

The authors declare no conflicts of interest.

### Peer Review

The peer review history for this article is available at https://publons.com/publon/10.1002/eji.202451386

## Supporting information



Supplementary information

## Data Availability

The data that support the findings of this study are available from the corresponding author upon reasonable request.
