# Supplementary material for: Similar Spatial Expression of Immune‐Related Proteins in SARS‐CoV‐2 Placentitis and Chronic Histiocytic Intervillositis
Source: Eur J Immunol. 2025 Jan 16;55(1):e202451386. doi: 10.1002/eji.202451386 (PMC11739671; doi:10.1002/eji.202451386)
Supplement: Supplementary file 1 — Supplementary information [file EJI-55-e202451386-s001.pdf]

SUPPLEMENTAL FILE

SARS-CoV-2 placentitis and chronic histiocytic intervillositis: distinct spatial expression of immune-related proteins

Michelle Broekhuizen[1,2], Marie-Louise van der Hoorn[3], Disha Vadgama[4], Michael Eikmans[5], Bojou J. Neecke[6], Johannes J. Duvekot[7], Pieter Fraaij[8,9], Irwin K.M. Reiss[1], Dana A.M. Mustafa[4], Lotte E. van der Meeren[4,10]*, Sam Schoenmakers[7]*

[1] Division of Neonatology, Department of Neonatal and Pediatric Intensive Care, Erasmus MC, Rotterdam, the Netherlands;

[2] Division of Pharmacology and Vascular Medicine, Department of Internal Medicine, Erasmus MC, Rotterdam, the Netherlands;

[3] Department of Obstetrics and Gynecology, Leiden University Medical Center, Leiden, the Netherlands;

[4] Department of Pathology, Erasmus MC University Medical Center, Rotterdam, the Netherlands;

[5] Department of Immunology, Leiden University Medical Center, Leiden, Netherlands;

[6] Department of Pathology, Pathan, Rotterdam, the Netherlands;

[7] Department of Obstetrics and Gynecology, Erasmus MC, Rotterdam, the Netherlands;

[8] Department of Viroscience, Erasmus MC, Rotterdam, The Netherlands;

[9] Division Infectious Diseases and Immunology, Department of Pediatrics, Erasmus MC, Rotterdam, the Netherlands;

[10] Department of Pathology; Leiden University Medical Center, Leiden, the Netherlands.

* These authors share last authorship

Supplemental Table S1. Clinical descriptives of the study populations.

Variable	SARS-CoV-2	CHI	Control
**N**	9	9	9
**Gestational age (weeks^{+days})**	29^{+1} (21^{+6} - 33^{+3})	33^{+3} (28^{+3} - 39^{+1})	37^{+5} (35^{+6} - 40^{+3})
**Fetal distress**	7 (78%)	4 (44%)	0 (0%)
**Fetal demise**	2 (22%)	0 (0%)	0 (0%)
**Fetal growth restriction**	0 (0%)	5 (44%)	0 (0%)
**Mode of delivery**			
Vaginal	2 (22%)	3 (33%)	1 (11%)
Elective cesarean section	0 (0%)	6 (67%)	8 (89%)
Emergency cesarean section	7 (78%)	0 (0%)	0 (0%)
**Placental disk weight (g)**	308 (192 - 450)	278 (144 - 392)	449 (375 - 516)

Data are depicted as median (range) or N (%).

Supplemental Table S2. Included proteins in the GeoMx® Digital Spatial Profiler assay.

GeoMx® Protein Assay	Protein	Full protein name	Group membership(s)
Immune cell profiling	Ms IgG2a		Negative control
Immune cell profiling	Ms IgG1		Negative control
Immune cell profiling	Rb IgG		Negative control
Immune cell profiling	Histone H3	Histone H3	Housekeeping control
Immune cell profiling	S6	Ribosomal protein S6	Housekeeping control
Immune cell profiling	GAPDH	Glyceraldehyde-3-phosphate dehydrogenase	Housekeeping control
Immune cell profiling	Beta-2-microglobulin / B2M	Beta-2-microglobulin	Nucleated cells
Immune cell profiling	CD11c / ITGAX	Integrin subunit alpha X	Myeloid: dendritic cell
Immune cell profiling	CD20 / MS4A1	Membrane spanning 4-domains A1	B cells
Immune cell profiling	CD3	CD3 T-cell receptor complex	T cells
Immune cell profiling	CD4	CD4 molecule	T cells: Th cells, myeloid
Immune cell profiling	CD45 / PTPRC	Protein tyrosine phosphatase receptor type C	Total immune
Immune cell profiling	CD56 / NCAM1	Neural cell adhesion molecule 1	NK cells
Immune cell profiling	CD68	CD68 molecule	Myeloid: macrophage
Immune cell profiling	CD8	CD8 molecule	T cells: CD8 T cells
Immune cell profiling	CTLA4	Cytotoxic T-lymphocyte associated protein 4	T cells: T cell activation, immune checkpoint
Immune cell profiling	Fibronectin	Fibronectin	Stroma: fibroblasts
Immune cell profiling	GZMB	Granzyme B	Cytotoxicity
Immune cell profiling	HLA-DR	Major histocompatibility complex, class II, DR	Antigen presentation: MHC class II
Immune cell profiling	Ki-67 / MKI67	Marker of proliferation Ki-67	Proliferation
Immune cell profiling	PanCk	Pan-cytokeratin	Epithelial, tumor
Immune cell profiling	PD-1 / PDCD1	Programmed cell death 1	T cells: T cell activation, immune checkpoint
Immune cell profiling	PD-L1	CD274 molecule	Myeloid: myeloid activation, immune checkpoint
Immune cell profiling	SMA	Smooth muscle actin	Stroma: smooth muscle cells
Immune cell typing	CD14	CD14 molecule	Myeloid: monocyte
Immune cell typing	CD163	CD163 molecule	Myeloid: macrophage, M2 macrophage

Table continued on next page

Supplemental Table S2. Continued

GeoMx® Protein Assay	Protein	Full protein name	Group membership(s)
Immune cell typing	CD34	CD34 molecule	Hematopoietic
Immune cell typing	CD45RO	CD45 leukocyte common antigen, 180 kDa isoform	Immune memory, T cells
Immune cell typing	CD66b / CEACAM8	CEA cell adhesion molecule 8	Myeloid: neutrophil
Immune cell typing	FAP-alpha / FAP	Fibroblast activation protein alpha	Stroma: fibroblasts
Immune cell typing	FOXP3	Forkhead box P3	T cells: Tregs, Th cells
Immune activation status	CD127 / IL7R	Interleukin 7 receptor	T cells: naive and memory
Immune activation status	CD25 / IL2RA	Interleukin 2 receptor subunit alpha	T cells: Tregs, T cell Activation
Immune activation status	CD27	CD27 molecule	T cells: T cell Activation
Immune activation status	CD40	CD40 molecule	Myeloid: myeloid activation
Immune activation status	CD44	CD44 molecule	Cell adhesion
Immune activation status	CD80	CD80 molecule	Myeloid: myeloid activation
Immune activation status	ICOS	Inducible T cell costimulator	T cells: T cell activation
Immune activation status	PD-L2	Programmed cell death 1 ligand 2	Antigen presentation: immune checkpoint, T cells
Cell death	BAD	BCL2 associated agonist of cell death	Apoptosis: pro-apoptosis
Cell death	BCL6	BCL6 transcription repressor	Apoptosis: anti-apoptosis
Cell death	BCLXL / BCL2L1	BCL2 like 1	Apoptosis: anti-apoptosis
Cell death	BIM / BCL2L11	BCL2 like 11	Apoptosis: pro-apoptosis
Cell death	CD95 / Fas	Fas cell surface death receptor	Apoptosis: pro-apoptosis
Cell death	Cleaved Caspase 9	Cleaved caspase 9	Apoptosis: caspase
Cell death	GZMA	Granzyme A	Cytotoxicity
Cell death	NF1	Neurofibromin 1	Tumor Suppressor
Cell death	p53	Tumor protein p53	Tumor Suppressor, DNA Damage
Cell death	PARP / PARP1	Poly(ADP-ribose) polymerase 1	DNA damage
